# Effect of Milk Type on the Microbiological, Physicochemical and Sensory Characteristics of Probiotic Fermented Milk

**DOI:** 10.3390/microorganisms7090274

**Published:** 2019-08-21

**Authors:** Dimitra Dimitrellou, Chrysoula Salamoura, Artemis Kontogianni, Despoina Katsipi, Panagiotis Kandylis, George Zakynthinos, Theodoros Varzakas

**Affiliations:** 1 Department of Chemistry, University of Patras, 26504 Patras, Greece; 2 Department of Food Science and Technology, University of Peloponnese, Antikalamos, 24100 Kalamata, Greece; 3 Department of Food Science and Technology, School of Agriculture, Aristotle University of Thessaloniki, P.O. Box 235, 54124 Thessaloniki, Greece

**Keywords:** goat milk, cow milk, *Lactobacillus casei*, starter culture, sensory

## Abstract

The production of fermented milk using cow milk, goat milk and a mixture of them (1:1) was evaluated. The traditional yogurt starter culture was used—*Streptococcus thermophilus* and *Lactobacillus delbrueckii* ssp. *bulgaricus*, along with *Lactobacillus casei* ATCC 393. The objective was to monitor the viability of these cultures during the storage of fermented milk at 4 °C for up to 28 days. Storage significantly affected the viability of all microorganisms and especially *L. bulgaricus*. All microorganisms retained viable numbers higher than 7.5 log CFU g^−1^, even after 4 weeks of storage, which is important to confirm the probiotic character of the product. The use of goat milk significantly affected the pH and acidity of fermented milk. More specifically, the use of goat milk led to fermented milk with lower pH values and higher acidities than fermented milk with cow milk. The sensory evaluation ascertained the overall quality of fermented milk with a mixture of cow and goat milk, which scored similar values to the commercial sample.

## 1. Introduction

Currently, there is a growing interest in the development of novel dairy products by substituting cow milk (CM) with milk from other origins. For example, a number of studies are using goat milk (GM) for dairy products such as yogurt, beverage, cheese, frozen dessert and ice cream [1,2,3,4]. This trend may be attributed to the nutritional, health and therapeutic benefits related with GM consumption [5]. GM has been characterized as having lower allergic potential compared to CM [6] and this may be attributed to better digestibility [7] due to differences in the amounts and structure of whey proteins [8] and the smaller size of fat globules [9]. Furthermore, the consumption of GM products has been correlated with better Cu intestinal absorption compared to cow products [10]. From a technological point of view, the smaller size of fat globules provides a smoother texture in derived GM products, the lower amounts of α_s1_-casein result in softer gel products, higher water-holding capacity and lower viscosity [11,12]. However, the main problem with the acceptance of dairy products based on GM is their intense taste and aroma compared to traditional dairy products made from CM [13]. Therefore, the use of GM in combination with CM may be a solution to successfully introduce GM in the market [11].

Another interest of the food industry is to develop innovative dairy products containing probiotic microorganisms, with a human health potential [14,15]. Dairy products and especially fermented milk (FMs) are ideal carriers for the delivery of probiotic bacteria in the gastrointestinal tract. However, the challenge in the preparation of such a probiotic delivery vehicle is to ensure a sufficient number of viable cells until the time of consumption [16]. *Lactobacillus casei* is among some of the most studied species due to its commercial, industrial and applied health potential [17]. Several strains of *L. casei* are considered to be probiotics. Among them, *L. casei* ATCC 393 is a well-known probiotic strain with many applications in food production [18,19,20,21].

Although the works concerning the production of dairy products by replacing CM with GM are sufficient [1,7,12,13], the majority of those do not focus on the survival of starter cultures or adjunct probiotic cultures. Therefore, the aim of the present study is to develop a FM based on GM and its mixture with CM in order to reduce the intense flavor. In the present study, probiotic FM were prepared using commercial starters (CH-1) and probiotic strain *L. casei* ATCC 393. The effect of milk origin on microbiological, physicochemical and sensorial characteristics of FM over a period of 4 weeks storage at 4 °C was evaluated. The new product is expected to combine the probiotic properties of *L. casei* ATCC 393 (like the regulation of intestinal microbiota [22], tumor-inhibitory, pro-apoptotic and anti-proliferative effects [23,24] and the production of bioactive peptides [18]) with the functional properties of GM (natural source of lactose-derived oligosaccharides, healthier lipid composition with increased conjugated linoleic acid and short fatty acids content, higher vitamin (A and complex B) and calcium content [6,25]).

## 2. Materials and Methods

### 2.1. Strains

The thermophilic starter CH-1 (Chr. Hansen, Hørsholm, Denmark) consisting of *Streptococcus thermophilus* and *Lactobacillus delbrueckii* spp. *bulgaricus* in freeze-dried form along with *Lactobacillus casei* ATCC 393 (DSM 20011; DSMZ, Braunschweig, Germany) were used in the present study. CH-1 culture was activated by adding a 50-U sachet to 500 mL of sterile skim milk (140 g L^−1^).

### 2.2. Fermented Milk Production

Pasteurized commercial milk (GM (fat 35 g L^−1^; sugars 45 g L^−1^; protein 36 g L^−1^) and CM (fat 35 g L^−1^; sugars 46 g L^−1^; protein 32 g L^−1^)) were heated at 37 °C and the activated CH-1 culture (0.3 % v/v; corresponding to 6 log CFU mL^−1^ *S. thermophilus* and 8 log CFU mL^−1^ *L. delbrueckii* spp. *bulgaricus*) and probiotic *L. casei* ATCC 393 (8 log CFU mL^−1^) were added. Milk was fermented at 37 °C until a pH value of 4.6 was reached. Thereafter, the FMs were cooled to 15 °C in ice water and then stored at 4 °C for up to 4 weeks. Three different FMs were produced, with the previously described procedure, using only CM, only GM and a mixture (1:1) of GM and CM.

### 2.3. Analyses

#### 2.3.1. Determination of Culture Viability

The viability of *S. thermophilus, L. bulgaricus and L. casei* was measured using FM samples of 10 g (diluted to sodium citrate solution (0.06 mol L^−1^)) and following the procedure described in a previous study [26,27]. More specifically, ¼ Ringer’s solution was used to prepare the appropriate serial dilutions. *Streptococcus thermophilus* was enumerated using M-17 agar supplemented with lactose at 45 °C, *L. bulgaricus* was enumerated using MRS agar, adjusted to pH 5.2 at 45 °C and *L. casei* was enumerated using lithium propionate MRS (LP-MRS) agar (MRS agar supplemented with lithium chloride (2 g L^−1^) and sodium propionate (3 g L^−1^)) at 37 °C. In addition, during storage, the potential growth of yeasts and molds (on malt agar at 30 °C for 72 h, pH was adjusted to 4.5 by sterile solution of 10% lactic acid) and coliforms (on violet red bile agar at 30 °C for 24 h) was monitored.

#### 2.3.2. Titratable Acidity and pH

A pH-meter was used for the determination of the pH of FM, while titration using 0.1 mol L^−1^ NaOH was used for the titratable acidity and expressed as g of lactic acid per 100 g of FM.

#### 2.3.3. Sensory Evaluation

The sensory evaluation was conducted by 20 panelists, familiar with the consumption of FM, using samples on the 14th day of storage. They were between 20 and 45 years of age, with 10 females and 10 males. All the samples (15 °C) were presented in uniform plastic cups, coded with random three-digit numbers. Water was provided between samples to cleanse the palate. The FMs were evaluated for color, sweet odor, sourness, smoothness, sweetness, viscosity, aftertaste and overall acceptability, using a 9-point hedonic scale ranging from 1 (“dislike extremely”) to 9 (“like extremely”). A commercial sample (yogurt from CM with 3.5% fat content) was also used for comparison. 

### 2.4. Experimental Design and Statistical Analysis

All experiments were carried out in triplicate. Significance was established at *P* < 0.05. The results were analyzed for statistical significance with ANOVA, and Tukey’s honest significant difference (HSD) test was used to determine significant differences between the results; coefficients, ANOVA tables, and significance (*P* < 0.05) were computed using Statistica version 5.0 (StatSoft Inc., Tulsa, OK, USA).

## 3. Results and Discussion

CM, GM and a mixture of them (1:1) were used in order to evaluate the effect of milk origin on the main characteristics of the new products. The mixture of CM and GM (1:1) was selected, since this combination is more acceptable by the consumers [12,13,28].

### 3.1. Physicochemical Characteristics

The storage time significantly affected titratable acidity and pH (*P* < 0.05) in all cases (Table 1 and Table 2). A continuous increase in titratable acidity was detected, while pH was reduced during storage, in correlation with a previous study using spray-dried *L. casei* ATCC 393 cells [26]. During storage, FM with GM presented significantly lower pH and higher acidity, followed by the FM with the mixture of milks. These results suggest that GM addition led to higher acidification, which is in accordance with previous studies using GM or a combination of GM and CM [11,12,28]. The use of different types of milk may affect the acidification rate of different lactic acid bacteria and therefore some of them may be more active in CM while others may be more active in GM [28]. For example, in GM, *L. delbrueckii* ssp. *bulgaricus* presents enhanced microbial growth, acidity progress and peptidase activity [29]. During storage, post-acidification was observed in all FM, but it was higher in the case of GM. More specifically, after 4 weeks of storage, the acidity of the FM was 1.26 g/100g, 1.35 g/100 g and 1.45 g/100 g in the case of CM, GM and mixture of milks, respectively. This trend may be attributed to the lower buffering capacity of GM compared to CM [30].

### 3.2. Microbiological Characteristics

In the dairy industry, it is very important to facilitate the survival of probiotic cells during the processing and storage of dairy products. Although the works concerning the production of dairy products by replacing cow with GM are sufficient [1,7,12,13,28], the majority of those do not focus on the survival of starter cultures or adjunct probiotic cultures. In the present study, the effect of GM addition and the storage on the survival of starter (*S. thermophilus* and *L. bulgaricus*) and probiotic cultures (*L. casei* ATCC 393) was evaluated.

*Streptococcus thermophilus* retained high viable counts during the storage period (Table 3). In general, this microorganism has been proven as capable of surviving in sufficient numbers even at low temperatures [26,31,32]. No significant differences were observed between the samples with different types of milk. A similar observation has been reported in a previous study using CM, GM and camel milk, although in GM, its survival was lower [31].

The other microorganism of the starter culture, *L. bulgaricus*, demonstrated the highest reduction in counts, during the storage period, especially in the case of GM (Table 4). This result is not in accordance with a previous study that presents enhanced microbial growth in GM [29]. However, in this study, *L. casei* is also present and may act antagonistically against *L. bulgaricus* [26]. However, the total sum of *S. thermophilus* and *L. bulgaricus* was above the minimum requirement of 7 log CFU viable microorganisms per g [33].

Regarding the viability of the probiotic strain added in the FM, storage affected (*P* < 0.05) its viability (Table 5). No significant differences were observed between the products with different types of milk, although lower values were observed after 4 weeks of storage in GM. In a previous study, using probiotics in GM beverage, the probiotics grew well and retained their viability during storage. However, the final pH of the product was high and cannot be compared with the low pH values of FM [34]. Of note, levels of the *L. casei* cells in the FM, during production and refrigerated storage (up to 28 days) remained above the minimum requirement (6 log CFU g^−1^) for adjunct cultures [33] and for probiotic foods, according to US FDA (United States Food and Drug Administration) and food industry recommendations [35], suggesting that the consumption of only 10–100 g of products may fulfill the daily intake of 8–9 log CFU of probiotic cells [36].

In general, the use of a GM and CM mixture for FM production did not significantly affect the viability of all microorganisms compared to CM. Finally, no yeasts, molds, or coliform bacteria were detected in any of the FM produced in the present study.

### 3.3. Sensory Evaluation

The most significant problem with the application of GM to several dairy products is its intense flavor compared to CM [2]. In addition, several studies showed that the use of GM affected the sensory characteristics of the FMs or yogurts, such as lower firmness, consistency, cohesiveness and viscosity [1]; lower hardness, adhesiveness, extension forces and higher susceptibility to syneresis [37]; lower firmness [12]. Therefore, the sensory analysis of the produced FMs (Table 6) is very important in order to identify acceptability by the consumers. Similar scores for color in all samples were reported apart from cow FM that receive significantly lower scores. This in accordance with previous studies where GM yogurts and cheeses were characterized as “whiter” compared to products with high levels of CM [7,12]. The color of FM is related with the appearance of the products and therefore with the acceptability by the consumers [13]. The score for aftertaste increased significantly (*P* < 0.05) when more GM was added, while the CM and commercial sample received lower scores. Similar results were observed in concentrated yogurt with GM [28] and may be attributed to the characteristic taste of GM products. There was a significant difference (*P* = 0.0014) in FMs for viscosity. FM with only GM received lower values followed by the mixture of GM and CM and then only CM and commercial sample, as also reported in a previous study with yogurts [13] and FM beverage [2]. These results may be attributed to lower casein content and a difference in casein proportions between GM and CM, leading to a more fragile clot and lower viscosity [38]. The use of different types of milk leads to different viscosity levels, with GM leading to lower values followed by CM, while sheep milk leads to higher values [37]. In terms of overall acceptability, there were two groups of products. The first group that received lower scores was GM and CM and the second group with higher scores was the mixture of GM and CM and the commercial sample. These results show the great potential for industrial application of the technology of mixed CM and GM products, due to the acceptance during sensory evaluation.

## 4. Conclusions

Microbiological analysis of fermented milk revealed that the use of different milk types does not affect the viability of *Streptococcus thermophilus* and probiotic *L. casei* ATCC 393, but only that of *Lactobacillus delbrueckii* spp. *bulgaricus*. The use of goat milk lead to significantly lower pH values. The sensory evaluation ascertained the overall quality of fermented milk with the mixture of cow and goat milk that scored similar values to the commercial sample. These results indicated that the use of mixture (1:1) goat and cow milk for fermented milk production has a great potential for the introduction of goat milk to industrial use, minimizing the intense flavor. Finally, the combination of probiotic properties of *L. casei* with the functional characteristics of goat milk might have a great potential for industrial application for the production of novel functional dairy products.

## Figures and Tables

**Table 1:** Effect of storage on pH values of fermented milk produced using goat and cow milk and their mixture.

Days	Goat Milk	Cow Milk	Mixture of Goat/Cow Milk
pH			
7	4.13 ± 0.02 ^a,A^	4.31 ± 0.01 ^a,B^	4.31 ± 0.01 ^a,B^
14	3.94 ± 0.03 ^b,A^	4.22 ± 0.05 ^a,B^	4.01 ± 0.01 ^b,A^
21	3.85 ± 0.03 ^b,A^	4.00 ± 0.03 ^b,A^	3.96 ± 0.02 ^bc,A^
28	3.83 ± 0.03 ^b,A^	3.98 ± 0.04 ^b,A^	3.88 ± 0.02 ^c,A^

^a–c^ Means within a column with different lowercase superscripts differ significantly (*P* < 0.05); ^A–B^ Means within a row with different uppercase superscripts differ significantly (*P* < 0.05).

**Table 2:** Effect of storage on acidity (g of lactic acid per 100 g of fermented milk) of fermented milk produced using goat and cow milk and their mixture.

Days	Goat Milk	Cow Milk	Mixture of Goat/Cow Milk
Titratable Acidity (g of Lactic Acid per 100 g of Fermented Milk)			
7	1.04 ± 0.01 ^a,A^	0.88 ± 0.03 ^a,B^	1.05 ± 0.01 ^a,A^
14	1.26 ± 0.04 ^b,A^	1.07 ± 0.03 ^b,B^	1.10 ± 0.01 ^a,B^
21	1.44 ± 0.02 ^c,A^	1.19 ± 0.02 ^bc,B^	1.25 ± 0.02 ^b,B^
28	1.45 ± 0.03 ^c,A^	1.26 ± 0.03 ^c,B^	1.35 ± 0.03 ^b,AB^

^a–c^ Means within a column with different lowercase superscripts differ significantly (*P* < 0.05); ^A–B^ Means within a row with different uppercase superscripts differ significantly (*P* < 0.05).

**Table 3:** Survival (log CFU g^−1^) of *Streptococcus thermophilus* in fermented milk during refrigerated storage at 4 °C.

Days	Goat Milk	Cow Milk	Mixture of Goat/Cow Milk
0	8.53 ± 0.07 ^a,A^	8.56 ± 0.03 ^a,A^	8.44 ± 0.05 ^a,A^
7	8.40 ± 0.04 ^ab,A^	8.34 ± 0.01 ^b,A^	8.31 ± 0.01 ^ab,A^
14	8.29 ± 0.02 ^ab,A^	8.22 ± 0.06 ^b,A^	8.30 ± 0.01 ^ab,A^
21	8.34 ± 0.05 ^ab,A^	8.20 ± 0.01 ^bc,A^	8.24 ± 0.05 ^ab,A^
28	8.15 ± 0.06 ^b,A^	8.02 ± 0.03 ^c,A^	8.15 ± 0.08 ^b,A^

^a–c^ Means within a column with different lowercase superscripts differ significantly (*P* < 0.05); ^A^ Means within a row with different uppercase superscripts differ significantly (*P* < 0.05).

**Table 4:** Survival (log CFU g^−1^) of *Lactobacillus delbrueckii* spp. *bulgaricus* in fermented milk during refrigerated storage at 4 °C.

Days	Goat Milk	Cow Milk	Mixture of Goat/Cow Milk
0	8.57 ± 0.06 ^a,A^	8.58 ± 0.07 ^a,A^	8.57 ± 0.05 ^a,A^
7	8.13 ± 0.03 ^b,A^	8.31 ± 0.02 ^b,B^	8.32 ± 0.01 ^b,B^
14	8.00 ± 0.03 ^bc,A^	8.25 ± 0.03 ^bc,B^	8.28 ± 0.02 ^b,B^
21	7.82 ± 0.05 ^cd,A^	8.01 ± 0.02 ^cd,A^	8.03 ± 0.03 ^c,A^
28	7.63 ± 0.04 ^d,A^	7.96 ± 0.06 ^d,B^	7.91 ± 0.01 ^c,B^

^a–d^ Means within a column with different lowercase superscripts differ significantly (*P* < 0.05); ^A–B^ Means within a row with different uppercase superscripts differ significantly (*P* < 0.05).

**Table 5:** Survival (log CFU g^−1^) of *Lactobacillus casei* ATCC 393 in fermented milk during refrigerated storage at 4 °C.

Days	Goat Milk	Cow Milk	Mixture of Goat/Cow Milk
0	8.78 ± 0.05 ^a,A^	8.83 ± 0.04 ^a,A^	8.83 ± 0.02 ^a,A^
7	8.59 ± 0.02 ^ab,A^	8.55 ± 0.08 ^ab,A^	8.72 ± 0.03 ^a,A^
14	8.55 ± 0.01 ^abc,A^	8.45 ± 0.05 ^b,A^	8.50 ± 0.04 ^b,A^
21	8.41 ± 0.07 ^bc,A^	8.40 ± 0.02 ^b,A^	8.48 ± 0.01 ^bc,A^
28	8.24 ± 0.09 ^c,A^	8.43 ± 0.06 ^b,A^	8.31 ± 0.04 ^c,A^

^a–c^ Means within a column with different lowercase superscripts differ significantly (*P* < 0.05); ^A^ Means within a row with different uppercase superscripts differ significantly (*P* < 0.05).

**Table 6:** Sensory evaluation of fermented milk produced using goat and cow milk and their mixture in comparison with commercial sample.

Sensory Attribute	Goat Milk	Cow Milk	Mixture of Goat/Cow Milk	Commercial
Color	8.3 ± 0.1 ^a^	7.1 ± 0.1 ^b^	8.2 ± 0.1 ^a^	8.3 ± 0.1 ^a^
Sweet Odor	5.5 ± 0.2 ^a^	5.1 ± 0.1 ^a^	5.6 ± 0.1 ^a^	7.0 ± 0.1 ^b^
Aftertaste	7.2 ± 0.1 ^a^	5.9 ± 0.1 ^c^	6.6 ± 0.1 ^b^	6.2 ± 0.1 ^bc^
Viscosity	6.8 ± 0.1 ^a^	8.4 ± 0.1 ^c^	7.5 ± 0.1 ^b^	8.0 ± 0.1 ^bc^
Sweetness	4.5 ± 0.1 ^a^	4.9 ± 0.1 ^b^	5.5 ± 0.1 ^b^	6.1 ± 0.1 ^c^
Smoothness	8.3 ± 0.1 ^ab^	7.4 ± 0.1 ^a^	8.5 ± 0.2 ^b^	7.8 ± 0.2 ^ab^
Sourness	5.9 ± 0.1 ^a^	7.1 ± 0.1 ^b^	5.7 ± 0.2 ^a^	6.0 ± 0.2 ^a^
Overall acceptability	7.9 ± 0.1 ^a^	7.8 ± 0.1 ^a^	8.7 ± 0.1 ^b^	8.6 ± 0.1 ^b^

^a–c^ Means within a row with different superscripts differ significantly (*P* < 0.05).
